# Complete Genome Sequence of the *Crocuta crocuta* Papillomavirus Type 1 (CcrPV1) from a Spotted Hyena, the First Papillomavirus Characterized in a Member of the *Hyaenidae*

**DOI:** 10.1128/genomeA.00062-12

**Published:** 2013-01-15

**Authors:** Hans Stevens, Elisabeth Heylen, Karlien De Keyser, Roger Maes, Matti Kiupel, Annabel Wise, Keith Nelson, Kay Holekamp, Anne Engh, Christy McKnight, Marc Van Ranst, Annabel Rector

**Affiliations:** a Laboratory of Clinical and Epidemiological Virology, Department of Microbiology and Immunology, Rega Institute for Medical Research, KU Leuven, Leuven, Belgium; b Diagnostic Center for Population and Animal Health, Michigan State University, East Lansing, Michigan, USA; c Pathology Department, MPI Research, Mattawan, Michigan, USA; d Department of Zoology, Michigan State University, East Lansing, Michigan, USA; e Department of Biology, Kalamazoo College, Kalamazoo, Michigan, USA; f Diagnostic and Toxicological Pathology Consultant, Kalamazoo, Michigan, USA

## Abstract

We report the complete genomic sequence of the *Crocuta crocuta* papillomavirus type 1 (CcrPV1), isolated from an oral papillomatous lesion of a wild spotted hyena. This virus is the first papillomavirus found in a species belonging to the *Hyaenidae* family of carnivores, and it can be classified in the genus *Lambdapapillomavirus*.

## GENOME ANNOUNCEMENT

Papillomaviruses (PVs) are a large family of small nonenveloped double-stranded DNA viruses that can cause benign and malignant proliferations of the stratified squamous epithelium of the skin and mucosa in a wide variety of vertebrate species. They are highly species specific, or at least restricted to infection of closely related animal species. The genus *Lambdapapillomavirus* exclusively contains PVs of carnivore hosts belonging to the *Felidae* (FdPV1, LrPV1, PcPV1, PlpPV1 and UuPV1), *Canidae* (CPV1 and CPV6), and *Procyonidae* (PlPV1) (1). Phylogenetic analysis of the PVs in this genus demonstrated that the evolutionary history of the feline PVs is closely linked to that of their feline hosts, providing an estimate of the papillomaviral evolutionary rate by applying host-fossil calibrations (2). We now report the sequence of a novel *Lambdapapillomavirus*: the *Crocuta crocuta* PV type 1 (CcrPV1), the first PV of a species of the *Hyaenidae* family of the *Feliformia* suborder of carnivores.

Biopsy material was obtained from an oral papilloma of a spotted hyena living in the Masai Mara Game Reserve (Kenya). The histological appearance of the biopsy material was suggestive of papillomaviral plaques. PCR and sequencing with the PV-specific degenerate primers FAP59/FAP64 and E1F2/E1R4 on extracted DNA from this lesion resulted in partial sequences of the E1 and L1 open reading frame (ORF) of a novel PV (3, 4). Primers for long template PCR were selected in these partial sequences, and overlapping long PCR fragments, together encompassing the complete viral genome, were generated with the Expand Long Template PCR system (Roche Diagnostics). Two fragments of approximately 3.5 and 6 kb were cloned (TOPO XL PCR cloning kit; Invitrogen) and the complete genomic sequence was determined using the EZ::TN <TET-1> insertion kit (Epicenter). Sequencing was performed on an ABI Prism 3100 genetic analyzer (Applied Biosystems), and sequences were assembled in SeqManII (DNASTAR).

The CcrPV1 genome counts 8,344 bp. It contains five early ORFs (E1, E2, E4, E6, and E7) and two late ORFs (L1 and L2), all located on the same strand of its double-stranded genome. In addition to the classical noncoding region between the early and late region (NCR1), an NCR2 is present between the end of E2 and the start of L2, which has been identified in all current members of the *Lambdapapillomavirus* genus (2, 4–7). Classification of PVs is based on sequence similarity in the L1 gene, the most conserved genomic region (8). With an L1 nucleotide sequence identity of 70.7% to the closest related type, CPV6, CcrPV1 is classified as a new type in the *Lambdapapillomavirus* genus. This is confirmed by the clustering in a maximum-likelihood phylogenetic tree of the L1 sequences of CcrPV1 and 82 other PV types (type species of all PV genera and all known PV types from carnivore hosts; data not shown). Thus, phylogenetic analysis as well as the presence of the typical NCR2 demonstrates that CcrPV1 is evolutionarily related to the PVs of closely related host species within the *Lambdapapillomavirus* genus.

### Nucleotide sequence accession number. 

The genome sequence of CcrPV1 is available in GenBank under accession number HQ585856.
